# Aerobic Exercise Induces Functional and Structural Reorganization of CNS Networks in Multiple Sclerosis: A Randomized Controlled Trial

**DOI:** 10.3389/fnhum.2020.00255

**Published:** 2020-06-30

**Authors:** Jan-Patrick Stellmann, Adil Maarouf, Karl-Heinz Schulz, Lisa Baquet, Jana Pöttgen, Stefan Patra, Iris-Katharina Penner, Susanne Gellißen, Gesche Ketels, Pierre Besson, Jean-Philippe Ranjeva, Maxime Guye, Guido Nolte, Andreas K. Engel, Bertrand Audoin, Christoph Heesen, Stefan M. Gold

**Affiliations:** ^1^Institut für Neuroimmunologie und Multiple Sklerose, Universitätsklinikum Hamburg-Eppendorf, Hamburg, Germany; ^2^Klinik und Poliklinik für Neurologie, Universitätsklinikum Hamburg-Eppendorf, Hamburg, Germany; ^3^APHM, Hopital de la Timone, CEMEREM, Marseille, France; ^4^Aix Marseille Univ, CNRS, CRMBM, UMR 7339, Marseille, France; ^5^Institut und Poliklinik für Medizinische Psychologie, Universitätsklinikum Hamburg-Eppendorf, Hamburg, Germany; ^6^Universitäres Kompetenzzentrum für Sport—und Bewegungsmedizin (Athleticum), Universitätsklinikum Hamburg-Eppendorf, Hamburg, Germany; ^7^Department of Neurology, Medical Faculty, Heinrich-Heine University Düsseldorf, Düsseldorf, Germany; ^8^Department of Diagnostic and Interventional Neuroradiology, University Medical Center Hamburg Eppendorf, Hamburg, Germany; ^9^Department of Physiotherapy, University Medical Center Hamburg Eppendorf, Hamburg, Germany; ^10^Department of Neurophysiology and Pathophysiology, Universitätsklinikum Hamburg-Eppendorf, Hamburg, Germany; ^11^Charité—Universitätsmedizin Berlin, Freie Universität Berlin, Humboldt Universität zu Berlin, and Berlin Institute of Health (BIH), Klinik für Psychiatrie und Psychotherapie, Campus Benjamin Franklin (CBF), Berlin, Germany; ^12^Charité—Universitätsmedizin Berlin, Freie Universität Berlin, Humboldt Universität zu Berlin, and Berlin Institute of Health (BIH), Med. Klinik m.S. Psychosomatik, Campus Benjamin Franklin (CBF), Berlin, Germany

**Keywords:** CNS networks, neuroplasticity, exercise, multiple sclerosis, randomized controlled trial

## Abstract

**Objectives**: Evidence from animal studies suggests that aerobic exercise may promote neuroplasticity and could, therefore, provide therapeutic benefits for neurological diseases such as multiple sclerosis (MS). However, the effects of exercise in human CNS disorders on the topology of brain networks, which might serve as an outcome at the interface between biology and clinical performance, remain poorly understood.

**Methods**: We investigated functional and structural networks in patients with relapsing-remitting MS in a clinical trial of standardized aerobic exercise. Fifty-seven patients were randomly assigned to moderate-intensity exercise for 3 months or a non-exercise control group. We reconstructed functional networks based on resting-state functional magnetic resonance imaging (MRI) and used probabilistic tractography on diffusion-weighted imaging data for structural networks.

**Results**: At baseline, compared to 30 healthy controls, patients exhibited decreased structural connectivity that was most pronounced in hub regions of the brain. Vice versa, functional connectivity was increased in hubs. After 3 months, we observed hub independent increased functional connectivity in the exercise group while the control group presented a loss of functional hub connectivity. On a structural level, the control group remained unchanged, while the exercise group had also increased connectivity. Increased clustering of hubs indicates a better structural integration and internal connectivity at the top of the network hierarchy.

**Conclusion**: Increased functional connectivity of hubs contrasts a loss of structural connectivity in relapsing-remitting MS. Under an exercise condition, a further hub independent increase of functional connectivity seems to translate in higher structural connectivity of the whole brain.

## Introduction

Promoting neuroplasticity is an unmet need to counter neurodegeneration and disability in acute and chronic neurological diseases. Preclinical studies have provided accumulating evidence for the “neuroregenerative” potential of exercise e.g., by modulating molecular signaling pathways, neurogenesis, and cognitive performance in mice (Aguiar et al., 2011; Mattson et al., 2018). Moreover, exercise-induced synaptogenesis and neurogenesis correlate with the intensity of exercise and are detectable in different brain areas including the motor cortex, cerebellum, and the hippocampus (Biedermann et al., 2016; Gutierrez et al., 2018). First clinical studies have suggested that exercises may enhance cognitive performance—a putative clinical readout of neuroplasticity—in healthy individuals and patients with neurodegenerative diseases (Hillman et al., 2008; Hötting and Röder, 2013; Mak et al., 2017). These beneficial effects of exercise can be framed as an evolutionary scenario that enhances brain function and resilience of neurons (Mattson et al., 2018).

Based on these observations, exercise has received increasing attention as a putatively disease-modifying treatment for complex human CNS disorders. Multiple sclerosis (MS) is an inflammatory, demyelinating CNS disorder with a pronounced neurodegenerative component and can serve as a paradigmatic disease model of CNS network disruption (Reich et al., 2018). Indeed, some experimental evidence suggests that exercise can protect mice from MS-like disease (Klaren et al., 2014). In MS, a couple of small clinical trials have indicated that short-term exercise intervention can improve cognition, mobility, and other symptoms of MS (Motl and Gosney, 2008; Dalgas and Stenager, 2012; Briken et al., 2014; Heine et al., 2015; Motl et al., 2017; Kjølhede et al., 2018). However, the mechanisms of action of exercise on brain structure and function remain elusive.

Combining structural and functional magnetic resonance imaging (MRI) is a feasible method to investigate morphology, microstructure, and large scale organization of the human brain (Hötting and Röder, 2013; Hamaide et al., 2016; Oberlin et al., 2016; Suo et al., 2016; Kjølhede et al., 2018). Over the last decade, MRI based functional and structural network analyses have deepened our understanding of the complex organization of the human brain and they have been recommended as an outcome measure for brain diseases including MS (Kaiser, 2013; Griffa et al., 2013; Betzel et al., 2014; Fornito et al., 2015; Bede, 2017; Yuan et al., 2017). A fundamental feature is the hierarchical organization of neuronal networks in so-called hubs or rich-club nodes on one hand and peripheral nodes on the other (van den Heuvel and Sporns, 2011). Hubs have an essential managing and integrating role in the brain, show altered connectivity in several neurological diseases, and explain disability better than other MRI metrics (Achard et al., 2012; Collin et al., 2016; Daianu et al., 2016; Stellmann et al., 2017). Based on the combined evidence from preclinical research and the potential of network analysis to interrogate brain organization, we aimed to decipher how exercise impacts functional and structural CNS reorganization in MS and how such changes are related to the network topology (Tewarie et al., 2014; Enzinger et al., 2016; Rocca et al., 2016; Schoonheim, 2017).

## Materials and Methods

### Patients and Study Design

The AERCONN trial [Exercise in Multiple Sclerosis: Effects on Cognitive Function and Brain Connectivity (NCT02005237)] is a rater-blinded, 1:1 randomized, controlled trial of aerobic exercise training over 3 months in MS compared to a waitlist control group. Details on trial design, intervention, and clinical outcomes have been published before (Baquet et al., 2018). Briefly, we recruited patients with relapsing-remitting MS (RRMS) according to the *McDonald criteria 2010* (Polman et al., 2011) without relapse or disability progression during the last 3 months (January 2013–November 2015). A bicycle ergometer training was tailored to the individual level of physical fitness and consisted of 2–3 supervised sessions per week for 12 weeks. Out of 68 recruited patients, 58 had a complete baseline and follow-up assessment including cognition, fitness, and disability: EDSS (expanded disability status scale), SDMT (symbol digit modality test), VLMT (verbal learning and memory test), PASAT (Paced Auditory Serial Addition Test), PA (Phasic altertness), TA (Tonic altertness), Digit_bw (Digit backwards), Digit_fw (Digits forward), T25FW (timed 25 foot walk), 6MWT (6 min Walking Test), VO2_peak and VO2 max/kg. The study did not reach the primary endpoint to improve performance in the VLMT (Helmstädter et al., 2001), failed to improve other clinical endpoints but showed an improvement in physical fitness. A predefined explorative analysis of the trial was to investigate effects of aerobic exercise on functional and structural connectivity. To follow this aim, we pursued the previous hypothesis and methods developed in our group (Faivre et al., 2016; Stellmann et al., 2017). We predefined a blinded graph theoretical analysis of hub dependency as an outcome of interest. Data from 57 patients could be included as MRI assessments were missing in one patient. Thirty healthy controls were included as a reference from an observational cohort study with the same MRI assessments (flow diagram as Supplementary Material). Demographic data are summarized in Table 1. The ethics committee of the Hamburg Chamber of Physicians approved the trial (Registration Number PV4356) and all participants gave written consent before any testing under the protocol.

**Table 1:** Descriptive statistics.

	Waiting group *N* = 27	Exercise group *N* = 30	*p*-value	Healthy controls *N* = 30	*p*-value
Females *n* (%)	21 (77.8)	18 (60.0)	0.248^§^	20 (66.7)	0.353
Age years	40.0 (9.8)	38.3 (10.1)	0.530^#^	40.6 (8.7)	0.477
Disease duration years	5.3 (6.3)	6.1 (5.5)	0.646^#^
Right handed *n* (%)	25 (93.0)	28 (93.3)	0.259^§^	28 (93.3)	1.000
Education >12 years	21 (77.8)	24 (80.0)	0.897^§^	19 (63.3)	0.055
Non smokers *n* (%)	17 (63.0)	24 (80.0)	0.305^§^	24 (80.0)	0.293
Immunotherapy *n* (%)	11 (40.7)	19 (63.3)	0.150^§^
Disability
EDSS median (range)	1.5 (0–3.5)	1.5 (0–3.5)	0.574^§^
SDMT correct answers	58.0 (9.9)	60.0 (11.8)	0.491^#^
T25FW seconds	4.8 (0.8)	4.6 (0.8)	0.434^#^
6MWT meters	437 (78)	441 (107)	0.851^#^
FSMC score	49.4 (22.0)	49.3 (21.1)	0.990^#^
HADS Depression score	3.6 (3.6)	3.5 (3.3)	0.949^#^
Fitness
Weight kg	68.1 (10.6)	80.5 (19.9)	0.005^#^
VO_2_-peak ml kg^−1^ min^−1^	25.4 (5.2)	28.5 (7.5)	0.078^#^
Training sessions median (range)	0	22 (10–36)

*EDSS, Expanded Disability Status Scale (Global disability, range 0–10); SDMT, Symbol Digit Modality Test (Cognition); T25FW, Timed 25 Foot Walk (Mobility); 6MWT, 6-Minute Walking Test (Mobility); FSMC, Fatigue Scale for Motor and Cognitive Functions; HADS, Hospital Anxiety and Depression Scale; Data as mean (sd) if not otherwise indicated, § = Chi-Square-Test, # = Student’s t-Test*.

### MRI Data Acquisition and Processing

A detailed description of sequences and processing steps are available in the Supplementary Material. Briefly, MRI data were acquired with a 3T scanner and the protocol included T1/T2 weighted sequences, diffusion tensor imaging (DTI, 32 directions), and 10 min of resting-state activity as represented by the BOLD signal. We used T2 images for lesion mapping and applied the longitudinal stream (Reuter et al., 2012) in FreeSurfer software (Version 5.2.0) to extract 80 cortical regions per hemisphere for our structural and functional connectivity analyses. We used an established pipeline (Besson et al., 2014) to build individual structural networks based on whole-brain probabilistic fiber tracking with average FA along the tracts as edge weight. To reconstruct individual functional connectomes, we adapted an existing processing pipeline (Wirsich et al., 2016) by computing Pearson-correlation between each region’s wavelet coefficient time series, thresholding, and binarizing networks assuring better comparability of networks (Achard et al., 2012).

### Descriptive Statistics, Graph Metrics, and Hub Disruption Index

We performed descriptive statistics with a mean (SD) or median (range) corresponding to the nature of the data. We computed individual global and nodal graph metrics (igraph and tnet packages in R; Csardi and Nepusz, 2006; Opsahl, 2009). Global graph metrics of structural networks included global strength (i.e., the sum of edge weights in each network) as a measure of total connectivity in the network, the global clustering coefficient, and the average shortest path length (APL) between all nodes. For functional connectomes, we computed the clustering coefficient and APL. On a node level, we extracted strength, weighted betweenness and weighted clustering using the arithmetic mean method (Opsahl and Panzarasa, 2009) for structural networks. Functional node metrics included degree, global efficiency (Eglob), and local efficiency (Eloc; Achard et al., 2012).

The hub disruption index κ allows to investigate topology dependent network alterations and has originally been defined as the linear regression estimate between the mean degree of the nodes in controls (x-axis) and the difference between the mean degree of nodes in comatose patients and controls (y-axis; Achard et al., 2012). We followed this approach by defining the order of the nodes, which is the representation of the topology, according to the mean degree in functional networks of controls and similar by the strength of nodes in structural networks. κ^slope^ is the slope estimate and determines the topological dependency of abnormalities. κ^inter^ is the intercept of the regression estimate and represents a global increase or decrease in connectivity if κ^slope^ is not significant. For changes in the hub disruption index, we computed for each node the differences between baseline values and month 3 values (labeled as Δ^0–3^). To preserve a standardized topological representation overall analyses, controls’ degree and strength served always as reference. In addition to group-level analysis, we computed individual hub disruption indices by regressing individual nodal values against the reference values from controls.

### Statistics

For group comparison, we used either Student’s *t*-test or Chi-Square test. Differences between groups in the averaged hub disruption indices as well as changes over time were investigated with ANOVA. For individual data, we applied a linear mixed effect model with random intercepts to investigate group differences. Finally, we computed Pearson correlation coefficients to investigate the association between graph metrics and clinical data, respectively their changes. After false discovery rate correction, *p*-values below 0.05 were considered statistically significant.

## Results

### Markers of Global Network Organization

At baseline, MS patients showed lower global structural connectivity compared to healthy controls as measured by the total strength (*p* < 0.001, Supplementary Figure S1A). Structural clustering and APL, however, were similar between patients and controls (*p* = 0.924 and *p* = 0.500, respectively). In functional networks, patients had a higher clustering coefficient (*p* = 0.006) and a shorter APL (*p* = 0.038), both indicating a more densely connected network topology. However, these metrics did not change or differ between the exercise and waiting group at follow-up (Supplementary Figure S1B). We also observed no group differences for changes in lesion volume or total brain volume (Supplementary Figure S2).

### Topology Dependent Network Organization

**Figure 1:**
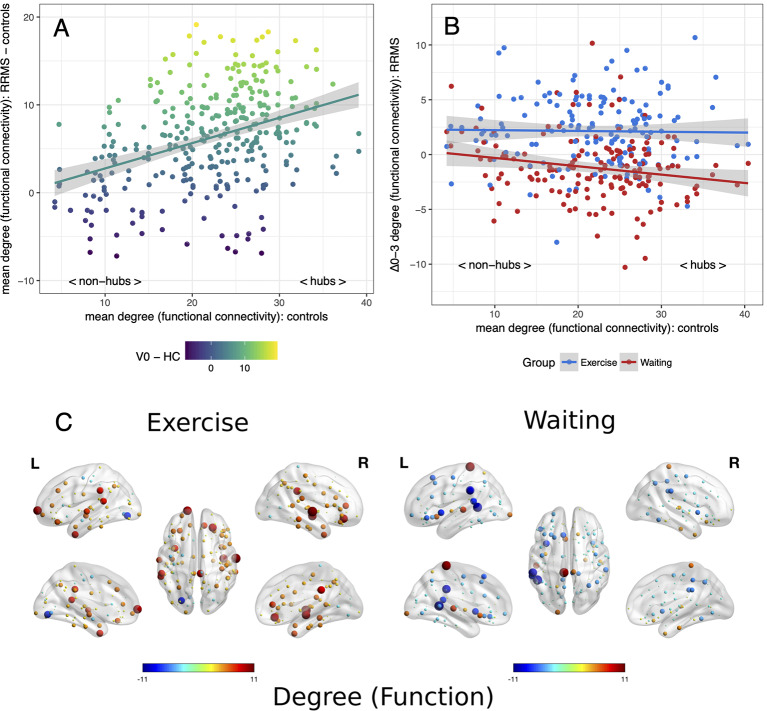
Reorganization of functional connectivity. Reorganization of functional connectivity based on the adapted hub disruption index. **(A)** Baseline: the mean degree of nodes from controls is plotted against the difference between mean baseline values from patients and controls. **(B)** Mean differences from baseline to month 3 (Δ0–3) in both patient groups are plotted against mean values from healthy controls. **(C)** Changes of a degree from baseline, node size indicates absolute change while the color indicates the direction.

**Figure 2:**
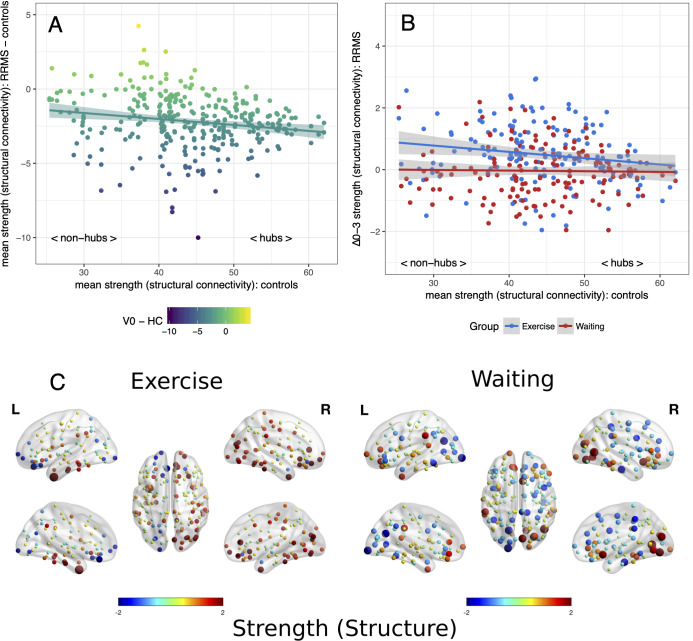
Reorganization of structural connectivity. Reorganization of structural connectivity based on the adapted hub disruption index. **(A)** Baseline: the mean strength of nodes from controls is plotted against the difference between mean baseline values from patients and controls. **(B)** Mean differences from baseline to month 3 (Δ0–3) in both patient groups are plotted against mean values from healthy controls. **(C)** Changes of strength from baseline, node size indicates absolute change while the color indicates the direction.

Applying the hub disruption index, we observed baseline differences between MS patients and controls indicating specific topological abnormalities in functional and structural connectomes in MS. Functional connectivity rose with ascending hubness of the nodes (κ^slope^ positive, *p* < 0.001, Figure 1A). Investigating structural connectomes, we observed an opposing effect with a pronounced loss of connectivity in hubs in MS (*p* < 0.001, Figure 2A).

Next, we examined the effects of exercise on these network parameters. Topology dependent changes in functional connectivity were observed between the waiting and the exercise group (time × group interaction *p* < 0.001, Figures 1B,C). A negative κ^slope^ (*p* = 0.018) indicated a decrease in hub connectivity in the waiting group. In contrast, the exercise group showed no differences between hubs and non-hubs (κ^slope^ = −0.01, *p* = 0.819) but a topology independent increase in connectivity (κ^inter^ = 2.30, *p* = 0.002). We observed similar results for structural connectivity with a group difference (time × group interaction *p* < 0.001): A topology independent increase of connectivity (κ^inter^ = 1.26, *p* = 0.003) was detected in the exercise group with somewhat less pronounced effects in hub regions (κ^slope^ = −0.02, *p* = 0.063). In contrast, we did not detect any significant changes from baseline for the waiting group (Figures 2B,C). Further graph metrics in functional networks provided less marked results for global and local efficiency of nodes (details are summarized in the Supplementary Material).

**Figure 3:**
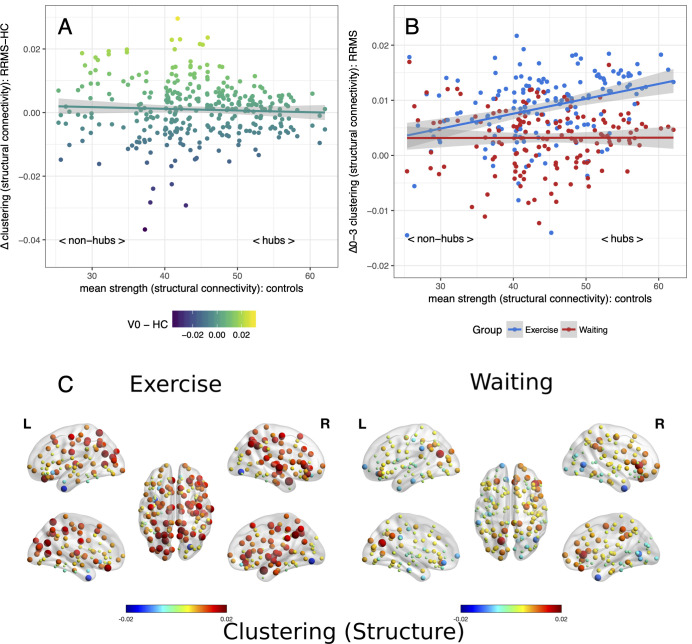
Clustering of nodes in structural networks: Baseline and changes. Nodal clustering in structural networks analyzed with the adapted hub disruption index. **(A)** Baseline: mean differences of the clustering coefficient between patients and controls plotted vs. strength of the nodes as an indicator of the hubness. **(B)** Mean differences from baseline to month 3 (Δ0–3) are plotted against mean strength from healthy controls for both patient groups. **(C)** Changes of clustering from baseline, node size indicates absolute change while the color indicates the direction.

The local clustering coefficient in structural networks is usually inversely correlated with the node hubness as found also in our cohorts (MS: *r* = −0.69, *p* < 0.001; Controls: *r* = −0.72, *p* < 0.001). At baseline, both patient groups showed an increased local clustering pronounced in non-hubs. After 3 months of exercise, we observed an increase in local clustering (κ^inter^ = 0.06, *p* < 0.001) that was accentuated in hubs (κ^slope^ = 0.07, *p* < 0.001) indicating a better structural integration and internal connectivity at the top of the network hierarchy. The control group did not show any changes from baseline (time × group interaction *p* < 0.001, Figure 3).

On an individual level, a group difference (*p* = 0.026) with a pronounced loss of node degree in hubs of the waiting control group compared to the exercise group confirmed group-level results for functional connectomes. No group differences were seen for the strength of nodes in structural connectomes (*p* = 0.345) while increased hub clustering in the exercise group compared to the control group was also confirmed (*p* = 0.007).

### The Association Between Functional and Structural Connectivity

To elucidate the association between functional and structural reorganization of networks, we investigated correlations between nodal graph metrics. Here, no associations were observed between changes in structural and functional metrics (Supplementary Results and Supplementary Figure S5). It should be noted that node metrics are also summary statistics and might not sufficiently account for the underlying reorganization within the network. To interrogate such effects, we extended our analysis to the edge level of the structural networks (see Supplementary Results and Supplementary Figure S6). We found the increase in connectivity to be accentuated in interhemispherical connections while deep gray matter connections seem to be unchanged.

To link structural and functional reorganization, we aimed to compare how efficient new functional connections are structurally wired. The length of the shortest structural path indicates the wiring costs between two nodes. Thus, we computed the differences in path lengths between baseline and month 3 for each pair of nodes. Overall, both groups had a gain in path length (*p* < 0.001), i.e., lower wiring costs. However, the gain was more pronounced in the exercise group (1.5% vs. 1.3%, *p* < 0.001).

### Association Between Network Metrics and Clinical Parameters

**Figure 4:**
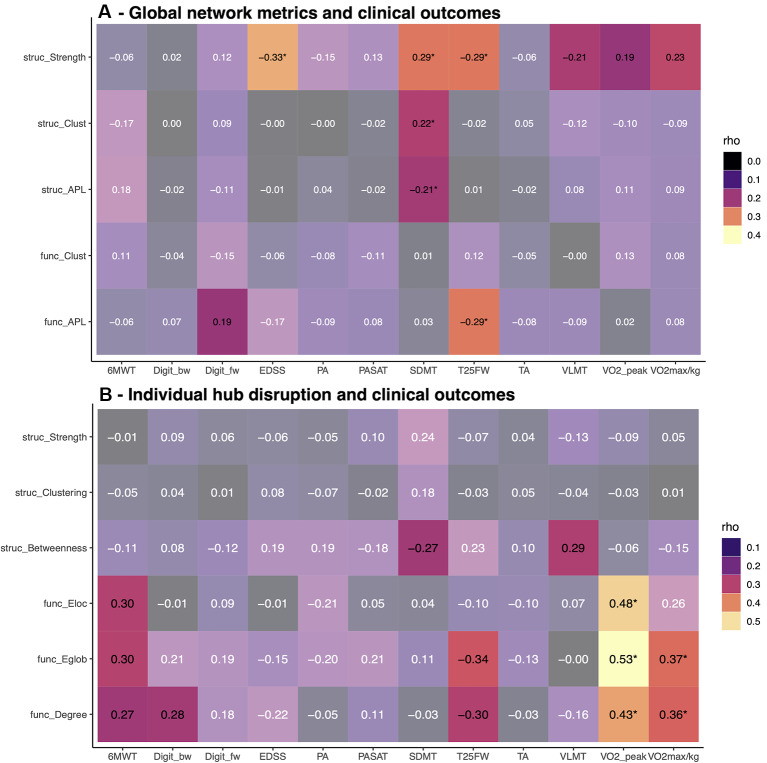
Association between graph metrics and clinical scores. Baseline correlations between clinical outcomes and global network metrics (top, **A**), respectively individual hub disruption (bottom, **B**). Bright colors indicate *p*-values < 0.05, asterisks indicate *p*-values below 0.05 after FDR correction. EDSS, expanded disability status scale; SDMT, symbol digit modality test; VLMT, Verbal learning and memory test; Digit_bw, digit span backward; Digit_fw, digit span forward; TA, tonic alertness; PA, phasic alertness; PASAT, Paced Auditory Serial Addition Test; T25FW = timed 25 foot walk; 6MWT, 6-Minute-Walking-Test; VO2max/kg, VO2 max per kg body weight.

The associations between clinical outcomes of disability, cognition, mobility, and fitness and network metrics at baseline are summarized in Figure 4. On a global level, we observed mainly associations of network strength with EDSS, SDMT, VLMT, T25FW, and physical fitness (Figure 4A). In contrast, individual κ^slope^ linked increased functional hub connectivity with higher fitness and walking tests at baseline (Figure 4B). This short-term study failed to reach the primary endpoint and only physical fitness increased in the exercise group over 3 months. Thus, we did not expect to find a strong association between clinical outcomes and network metrics. However, we observed weak associations between EDSS change and increased structural strength (rho = −0.28, *p* = 0.039) and shorter APL (rho = 0.29, *p* = 0.038). Out of the individual hub disruption indices, only higher betweenness of hubs was correlated with better VLMT performance (rho = 0.33, *p* = 0.013).

## Discussion

Besides clinical endpoints, the AERCONN study was designed to explore the impact of an exercise intervention on functional and structural connectivity in mildly disabled patients with RRMS. Aerobic exercise was chosen as the best available framework to investigate neuroplasticity (Hötting and Röder, 2013). We observed an increase of structural and functional connectivity induced by a moderate aerobic exercise training over 3 months. We were able to show that these network changes follow distinctive patterns that are associated with the fundamental hierarchical organization of the brain networks (van den Heuvel and Sporns, 2011). Moreover, exercise seems to increase the efficiency of new functional connections utilizing their structural wiring costs.

### Topology Dependent Alteration of Brain Networks in MS

Within recent years, investigating the topology of brain networks and their alterations in development, aging, neuroinflammatory and neurodegenerative diseases including MS has received increasing attention (Yuan et al., 2017). The most prominent nodes in these networks, which act as managing nodes with an important integrative role for the overall network performance are of special interest (Griffa et al., 2013; Collin et al., 2014; Tewarie et al., 2014; Baker et al., 2015; Daianu et al., 2016; Rocca et al., 2016; Li et al., 2017; Meijer et al., 2017; Stellmann et al., 2017; Wierenga et al., 2018). Our findings underline the feasibility of such an approach in clinical settings. Compared to healthy individuals, we observed a distinctive pattern of network alteration in MS patients at baseline, as the loss of structural connectivity was pronounced in hub regions. This observation is in line with a previous study in primary progressive MS from our group (Stellmann et al., 2017) and points towards a specific network pathology of MS. Interestingly, this alteration of the network topology is not detectable with global network metrics such as APL or global clustering—even though total structural connectivity is lower in MS. This discrepancy has been observed before and can be explained by the robustness of natural scale-free networks against local and diffuse attacks (Aerts et al., 2016; Stellmann et al., 2017). Metrics like APL are only altered if the large-scale organization is severely affected. For example on a functional level, even comatose patients do not show an alteration of global graph metrics in comparison to healthy controls (Achard et al., 2012).

A recent study also linked spatial lesion distributions in neurological diseases to a pronounced alteration of connectivity in the managing regions of the brain (Yuan et al., 2017). As MS lesions show a typical spatial distribution, a disease-specific pattern is not surprising. Trans-synaptic anterograde and retrograde neurodegeneration is a known pathophysiological process in MS (Gabilondo et al., 2014) and explains the promotion of connectivity loss in the top of the hierarchy independent from the actual distance between lesions and hubs.

The structural alteration was accompanied by increased functional connectivity in hub regions. The inverse relationship between structure and function might indicate a compensatory mechanism. However, increased connectivity has been described before as typical observation in MS (especially in early disease stages) and it remains controversial if such changes should be interpreted as adaptive or maladaptive (Audoin et al., 2003; Meijer et al., 2017; Schoonheim, 2017). While positive associations of clinical performance with higher activation levels might indicate a beneficial effect, others argue that high energy costs might be unfavorable given the importance of mitochondrial dysfunction and energy consumption in neurodegeneration (Lin and Beal, 2006; Campbell et al., 2014; Schoonheim, 2017). Here, we observed at baseline an association between increased functional hub connectivity and better physical fitness pointing towards a rather beneficial effect. However, the vast majority of data are cross-sectional and the need to investigate functional reorganization longitudinally has been emphasized (Schoonheim et al., 2015; Enzinger et al., 2016; Schoonheim, 2017).

### Topology Dependent Effects of Exercising on Brain Networks in MS

Our study provides insight into topology-dependent changes in structural and functional reorganization over 3 months. We observed a global increase in functional connectivity associated with a similar increase in the structural connectome under the exercise condition. An increased clustering of hub nodes indicated that such changes are reflecting the topology of network alterations observed at baseline. Thus, considering exercise as a beneficial and neuroprotective framework, increasing functional connectivity under-exercising seems rather reflect an adaptive mechanism than a maladaptive one.

In contrast to our findings, a recent cross-sectional study described a peripheral increase or non-hub increase in cognitively impaired patients, while cognitively preserved patients showed a similar pattern as controls (Meijer et al., 2017). The patients enrolled in our trial were still in the early disease stage, younger and less disabled, both physically and cognitively. The peripheral increase might, therefore, be explained as a feature of the later disease course where the adaptive increase in hub connectivity is exhausted. This explanation is supported by the observation, that our control group already showed a decrease in functional hub connectivity within 3 months. Moreover, a first longitudinal study observed an association between the loss of increased baseline connectivity and disability progression in MS (Faivre et al., 2016). Thus, one might hypothesize that increased functional connectivity represents a basic adaptive principle that only translates to sustained neuroplasticity on a molecular or neuronal level in a biophysiological context like exercising (Thomas et al., 2016).

Changes in structural connectivity in our exercise cohort represent primarily increased fractional anisotropy within the white matter, which is known to correlate with axonal density and myelinization and thus support our interpretation of exercise-induced neuroplasticity on a biological level (Mallik et al., 2014). The suspected mechanism of action is in line with previous findings that describe white matter microstructure, as assessed with diffusion tensor imaging, as an important mediator between physical fitness and cognition (Oberlin et al., 2016; Sexton et al., 2016). Finally, there is recent evidence that increased neuronal activity, as represented by increased functional connectivity, induces oligodendrogenesis, and adaptive remyelination that is furthermore associated with behavior (Gibson et al., 2014). The latter mechanism might also explain the gain in the path length of new functional connections that we observed in the waiting group.

## Limitations

Our study has several limitations. With only two visits, 3 months apart, our study cannot address whether structural changes follow functional reorganization or vice versa. As increased co-activation of brain regions can already be observed after a single session of exercising in healthy individuals (Perini et al., 2016), it seems reasonable to expect functional changes to occur before structural changes. Moreover, the short follow-up does not allow for conclusions about sustained neuroplasticity and long-term effects in a chronic disease such as MS. As our trial failed to show significant effects on clinical outcomes, our analyses were further restricted, as we were only able to detect weak associations between network changes and disability. Whether network adaptation can predict subsequent clinical improvement thus remains to be addressed in future trials. Moreover, our sample size was rather small, and as a result, we might have missed weak effects. Finally, without a healthy control group undergoing the same intervention, we were not able to distinguish between general and MS-specific effects of exercising on brain networks.

Taken together, we here provide evidence that increased functional connectivity of hubs seems to represent a fundamental adaptive mechanism of the brain to compensate for a loss of structural connectivity in neurological diseases. Moreover, regular exercise may provide a biophysiological framework that induces repair of disturbed structural connections in complex human brain disorders such as MS.

## Data Availability Statement

The datasets generated and analyzed during the current study are available from the corresponding author on reasonable request.

## Ethics Statement

The studies involving human participants were reviewed and approved by Ethics committee of the Hamburg Chamber of Physicians (Registration Number PV4356). The patients/participants provided their written informed consent to participate in this study.

## Author Contributions

J-PS, JP, K-HS, GN, AE, CH, and SMG have analyzed and interpreted data, drafted/revised the manuscript for content, and have contributed to the study concept and design. AM, PB, J-PR, MG, and BA have analyzed and interpreted data and have revised the manuscript for content. LB, SP, I-KP, SG, and GK have contributed to the study concept and design, the conduction of the trial and have revised the manuscript for content.

## Conflict of Interest

J-PS receives research funding from Deutsche Forschungsgemeinschaft and reports grants from Alexion, Biogen and Genzyme outside the submitted work. JP reports grants from Deutsche Rentenversicherung Bund outside the submitted work. I-KP has received honoraria for speaking at scientific meetings, serving at scientific advisory boards and consulting activities from Adamas Pharma, Almirall, Bayer Pharma, Biogen, Genzyme, Merck Serono, Novartis, and Teva. She has received research support from Merck Serono, Novartis, the German MS Society, and Teva. I-KP is the founder of the company COGITO GmbH, Zentrum für Angewandte Neurokognition und Neuropsychologische Forschung, Merowingerplatz 1, 40225 Düsseldorf, Germany. AE receives research funding from the Deutsche Forschungsgemeinschaft, Bundesministerium für Bildung und Forschung, and the EU. CH reports grants and personal fees from Biogen, personal fees from Genzyme, grants and personal fees from Novartis, grants from Merck Serono, outside the submitted work. SG reports honoraria from Mylan GmbH and Almirall S.A., research grants from Biogen, outside the submitted work. He receives research funding from the Deutsche Forschungsgemeinschaft, Bundesministerium für Bildung und Forschung, and the National MS Society. The remaining authors declare that the research was conducted in the absence of any commercial or financial relationships that could be construed as a potential conflict of interest.
