# Supplementary material for: Aerobic Exercise Induces Functional and Structural Reorganization of CNS Networks in Multiple Sclerosis: A Randomized Controlled Trial
Source: Front Hum Neurosci. 2020 Jun 30;14:255. doi: 10.3389/fnhum.2020.00255 (PMC7340166; doi:10.3389/fnhum.2020.00255)
Supplement: Supplementary file 1 [file Data_Sheet_1.PDF]

Aerobic exercise induces functional and structural reorganization of CNS networks in multiple sclerosis: A randomized controlled trial

Supplemental Material

Methods: MRI data acquisition and processing

Methods: Structural connectivity

Methods: Functional connectivity

Figure S1: Global graph metrics: Baseline and dynamics

Figure S2: Change of brain and T2 lesion volume

Topological reorganization: global efficiency and local efficiency in functional networks

Figure S3: Functional reorganization: Global efficiency

Figure S4: Functional reorganization: Local efficiency

The association between functional and structural connectivity

Figure S5: Correlation between node metrics

Patterns of connectivity changes in structural connectomes

Figure S6: Structural connectivity changes on the edge level

Sensitivity analysis: Thresholding resting state networks

MRI data acquisition and processing

MRI data were acquired with a 3T MRI scanner (Skyra, Siemens Medical Systems, Erlangen, Germany). The MRI protocol included the following sequences: a 3D magnetization prepared rapid acquisition gradient-echo (MPRAGE) T1 weighted sequence (TR/TE = 2500ms/2.12ms; TI=1100ms; 256 slices, voxel size 0.8×0.8×0.9 mm, no gap, matrix=288x288, FOV = 240mm), a T2 sequence (TR/TE = 2800ms/90ms; 43 slices, voxel size 0.5×0.5×3.0 mm, no gap, matrix=256x256, FOV = 240mm), diffusion tensor imaging (DTI, single-shell, 32 directions with non-collinear diffusion gradients (b=1000 s/mm2) and one non-diffusion-weighted (b=0 s/mm2), voxel size 1.9×1.9×2.0 mm, FOV 240 mm, matrix 128 x 128, all volumes averaged over three acquisitions, TR/TE = 7200ms/90ms; 54 slices, no gap). Resting state data consist of 250 functional MRI images acquired in a BOLD-sensitized EPI T2*-weighted sequence (TR/TE = 2500ms/25ms; TI=900ms; 40 slices, voxel size 2.7×2.7×3.0 mm, no gap, matrix=256x256, FOV = 250mm, FA=90°) summing up to 10 minutes of measuring time.

Images were processed with the functional imaging software library (FSL, version 5.0, www.fmrib.ox.ac.uk). 3D-sequences were reoriented to standard space and T1 images were registered to T2-weighted images for lesion mapping. T1-hypointense and T2-hyperintense lesions were outlined on T2 and registered T1 images with a seed-based semiautomatic algorithm using the software Analyze 11.0 (AnalyzeDirect, www.analyzedirect.com). To obtain MRI measurements commonly used in MS MRI studies for comparison, we extracted lesion volumes and used FSL SIENA to estimate percentage brain volume change from baseline to Month 3 (Smith et al., 2002). To extract reliable volume and thickness estimates, images where automatically processed with the longitudinal stream (Reuter et al., 2012) in FreeSurfer software (Version 5.2.0) for cortical reconstruction and volumetric segmentation (surfer.nmr.mgh.harvard.edu/). Specifically an unbiased within-subject template space and image was created using robust, inverse consistent registration. To minimize segmentation errors, we performed lesion filling on T1 weighted images by filling marked lesion areas with mean intensity values from the lesions' surrounding parenchyma prior to that. Moreover, we manually corrected brain masks and white / grey matter segmentation errors in all cases. For each individual subject and time point, we extracted the grey matter parcellation of 160 distinct regions (i.e, 80 regions per hemisphere) for our structural and functional connectivity analyses.

Structural connectivity

Next, we used an established pipeline (Besson et al., 2014) to build individual structural networks based on MRtrix3 (www.mrtrix.org) whole brain probabilistic fibre tracking. Summarized, DTI data were corrected for Eddy current distortion and head movement with the FSL diffusion imaging toolbox (Behrens et al., 2007). After fitting diffusion tensors at each voxel to calculate FA and mean diffusivity maps, the fibre orientation distribution (FOD) determined the response function. FOD accuracy was optimized by constrained spherical deconvolution (Tournier et al., 2007). A probabilistic tracking algorithm generated 150,0000 fibres of minimum length 20 mm (step size: 0.2 mm, minimum radius of curvature: 1mm, FOD cut-off: 0.1). The white matter mask was dilated by 1 mm into the cortex and each voxel of this mask served as potential seed and tracking continued until the mask boundary or another stopping criterion was reached (Tournier et al., 2012). Next, average FA for each fibre was computed after estimating FA values at each point of the fibre. To construct structural connectivity matrices, we

determined for every pair of grey matter regions *i* and *j*, if they were connected by at least one fibre. If so, the average FA along the tracts was used as edge weight or connectivity strength between *i* and *j*.

Functional connectivity

Again, we adapted an established processing pipeline to reconstruct individual functional connectomes for each time point (Wirsich et al., 2016). Briefly, we used SPM12 (www.fil.ion.ucl.ac.uk/spm/software/spm12) to correct BOLD resting state volumes for movements and slice timing as well as to coregister them with the T1 volume and the individual regional parcellation. We performed averaging of all voxels of each region and time point and regressed out head movement, cerebrospinal fluid signals, white matter signals and global mean signal. Applying wavelet analysis on the resulting region-averaged times series (brainwaver package in R) we only used wavelet coefficients of the third wavelet scale representing the frequency band from 0.1 Hz to 0.05 Hz for a TR of 2.5 s (Achard et al., 2012). We computed the raw functional connectivity matrix by calculating the Pearson-correlation between each region's wavelet coefficient time series. We did not differentiate between direct or inverse correlations, as both directions indicate a functional relation between regions. After correction for multiple testing with the false discovery rate, connectivity matrices were thresholded to generate binary networks including the top 15% of connections assuring a better comparability of networks (Achard et al., 2012). Additional sensitivity analyses at different thresholds are available in the supplemental material.

Methods: References

Achard, S., Delon-Martin, C., Vértes, P.E., Renard, F., Schenck, M., Schneider, F., Heinrich, C., Kremer, S., Bullmore, E.T., 2012. Hubs of brain functional networks are radically reorganized in comatose patients. Proc. Natl. Acad. Sci. U. S. A. 109, 20608–13. https://doi.org/10.1073/pnas.1208933109

Behrens, T.E.J., Berg, H.J., Jbabdi, S., Rushworth, M.F.S., Woolrich, M.W., 2007. Probabilistic diffusion tractography with multiple fibre orientations: What can we gain? Neuroimage 34, 144–155. https://doi.org/10.1016/j.neuroimage.2006.09.018

Besson, P., Dinkelacker, V., Valabregue, R., Thivard, L., Leclerc, X., Baulac, M., Sammler, D., Colliot, O., Lehéricy, S., Samson, S., Dupont, S., 2014. NeuroImage Structural connectivity differences in left and right temporal lobe epilepsy. Neuroimage 100, 135–144. https://doi.org/10.1016/j.neuroimage.2014.04.071

Reuter, M., Schmansky, N.J., Rosas, H.D., Fischl, B., 2012. Within-subject template estimation for unbiased longitudinal image analysis. Neuroimage 61, 1402–1418. https://doi.org/10.1016/j.neuroimage.2012.02.084

Smith, S.M., Zhang, Y., Jenkinson, M., Chen, J., Matthews, P.M., Federico, A., De Stefano, N., 2002. Accurate, robust, and automated longitudinal and cross-sectional brain change analysis. Neuroimage 17, 479–489. https://doi.org/10.1006/nimg.2002.1040

Tournier, J., Calamante, F., Connelly, A., 2012. MRtrix : Diffusion Tractography in Crossing Fiber Regions. https://doi.org/10.1002/ima.22005

Tournier, J.D., Calamante, F., Connelly, A., 2007. Robust determination of the fibre orientation distribution in diffusion MRI: Non-negativity constrained super-resolved spherical deconvolution. Neuroimage 35, 1459–1472. https://doi.org/10.1016/J.NEUROIMAGE.2007.02.016

Wirsich, J., Perry, A., Ridley, B., Proix, T., Golos, M., Bénar, C., Ranjeva, J., Bartolomei, F., Breakspear, M., Jirsa, V., Guye, M., 2016. NeuroImage : Clinical Whole-brain analytic measures of network communication reveal increased structure-function correlation in right temporal lobe epilepsy. YNICL 11, 707–718. https://doi.org/10.1016/j.nicl.2016.05.010

Figure S1: Global graph metrics: Baseline and dynamics

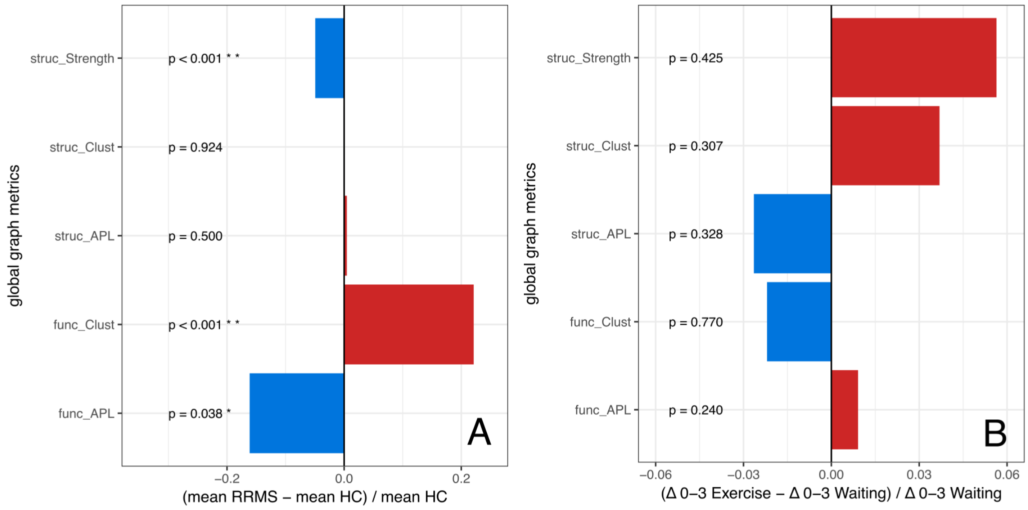

(A) Differences of global graph metrics between relapsing remitting MS (RRMS) and healthy controls (HC), func = functional connectivity, struc = structural connectivity, APL: average shortest path length, Clust: global clustering coefficient, Assort: assortativity, p-values from t-tests (* = $p<0.05$, ** = $p^{FDR}<0.05$) (B) Changes of global graph metrics as differences from baseline to month 3(Δ0-3) without significant differences between waiting and exercise group (ANOVA).

Figure S2: Change of brain and T2 lesion volume

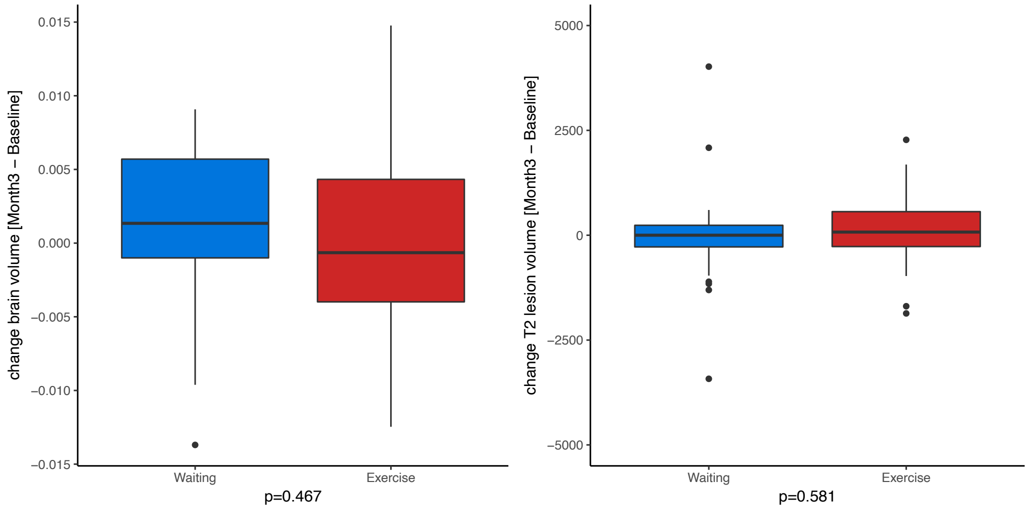

Changes of total brain volume (left) and T2 lesion volume (right) from baseline to month 3 without significant differences between waiting and exercise group.

Topological reorganization: global efficiency and local efficiency in functional networks

Investigating further graph metrics in functional networks, we observed comparable but less marked results for global and local efficiency of nodes as for the degree of nodes: The exercise and the control group differed concerning changes from baseline and we detected also a hub-independent increase of these node metrics in the exercise group after three months.

Similar to the degree of nodes, global efficiency was raised in patients if compared with controls at baseline and this effect increased with ascending hubness of the nodes (p<0.001, S3A). After three months, the change in global efficiency was larger in the exercise group (p<0.001) but did not differ between hubs and non-hubs (Figure S3B). Local efficiency was increased globally in patients at baseline (p=0.008) and the raises in local efficiency correlated inversely with the hubness of nodes (p<0.001), i.e. the effect was pronounced non-hub regions (Figure S4A). Groups differed in their changes of local efficiency after three months (time x group interaction p<0.001). Both groups presented a global increase in local efficiency independent from the hubness of the nodes, but the effect was accentuated in the exercise group (Figure S4B).

Figure S3: Functional reorganization: Global efficiency

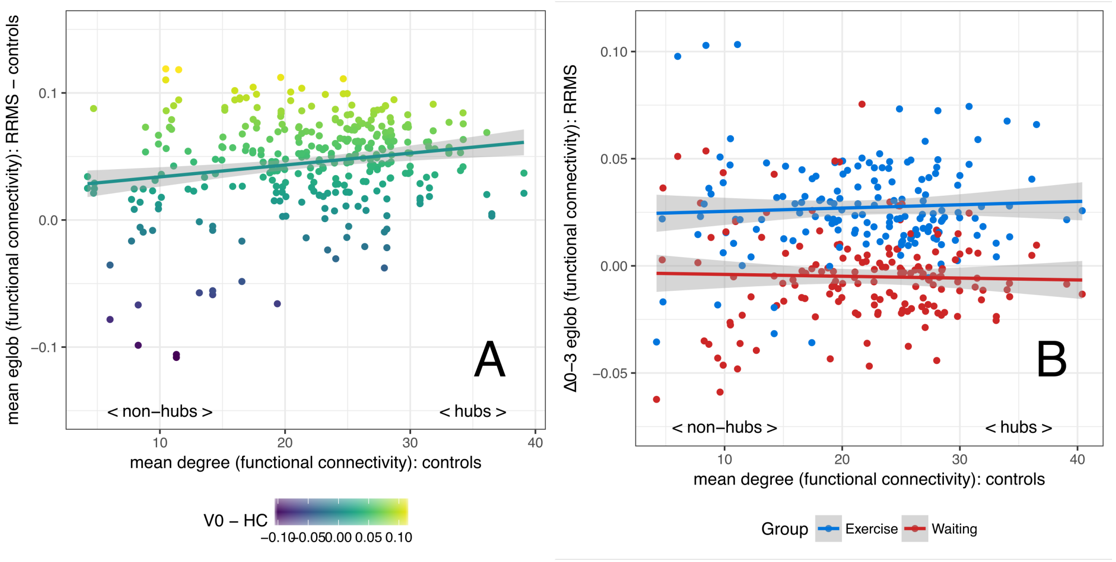

Adapted hub disruption index for global efficiency (eglob) in functional networks: (A) Baseline: Mean degree of nodes from controls is plotted against the difference between mean baseline eglob values from patients (B) Mean differences from baseline to month 3 (Δ0-3) are plotted against mean degree from healthy controls. RRMS = relapsing-remitting multiple sclerosis

Figure S4: Functional reorganization: Local efficiency

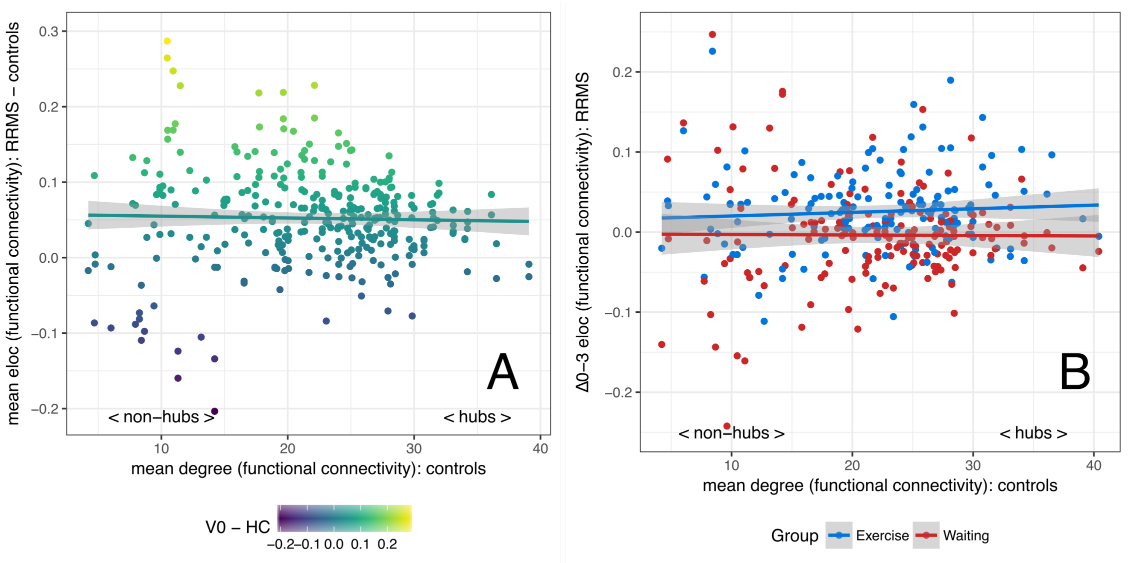

Adapted hub disruption index for local efficiency (eloc) in functional networks: (A) Baseline: Mean degree of nodes from controls is plotted against the difference between mean baseline eloc values from patients (B) Mean differences from baseline to month 3 (Δ0-3) are plotted against mean values from healthy controls. RRMS = relapsing-remitting multiple sclerosis

The association between functional and structural connectivity

To elucidate the association between functional and structural reorganization of networks, we first investigated correlations between graph metrics at both time points (supplemental figure S5) in controls and the two patient groups. We observed a moderate but homogenous correlation between the degree of nodes in functional networks and the strength of nodes in structural networks in controls (r=0.30, p<0.001) and patients (r=0.33, p<0.001). Thus, the hubness of nodes was comparable between functional and structural connectomes and not massively disrupted in comparison to controls. However, the cross-sectional correlations do not address if functional and structural connectivity changes are associated or independent from each other. Thus, we also performed correlation analyses between the changes of nodes' metrics from baseline to month 3. We observed no association between changes in structural and functional metrics (supplemental figure S5) and no difference between the two patients groups.

Figure S5: Correlation between node metrics

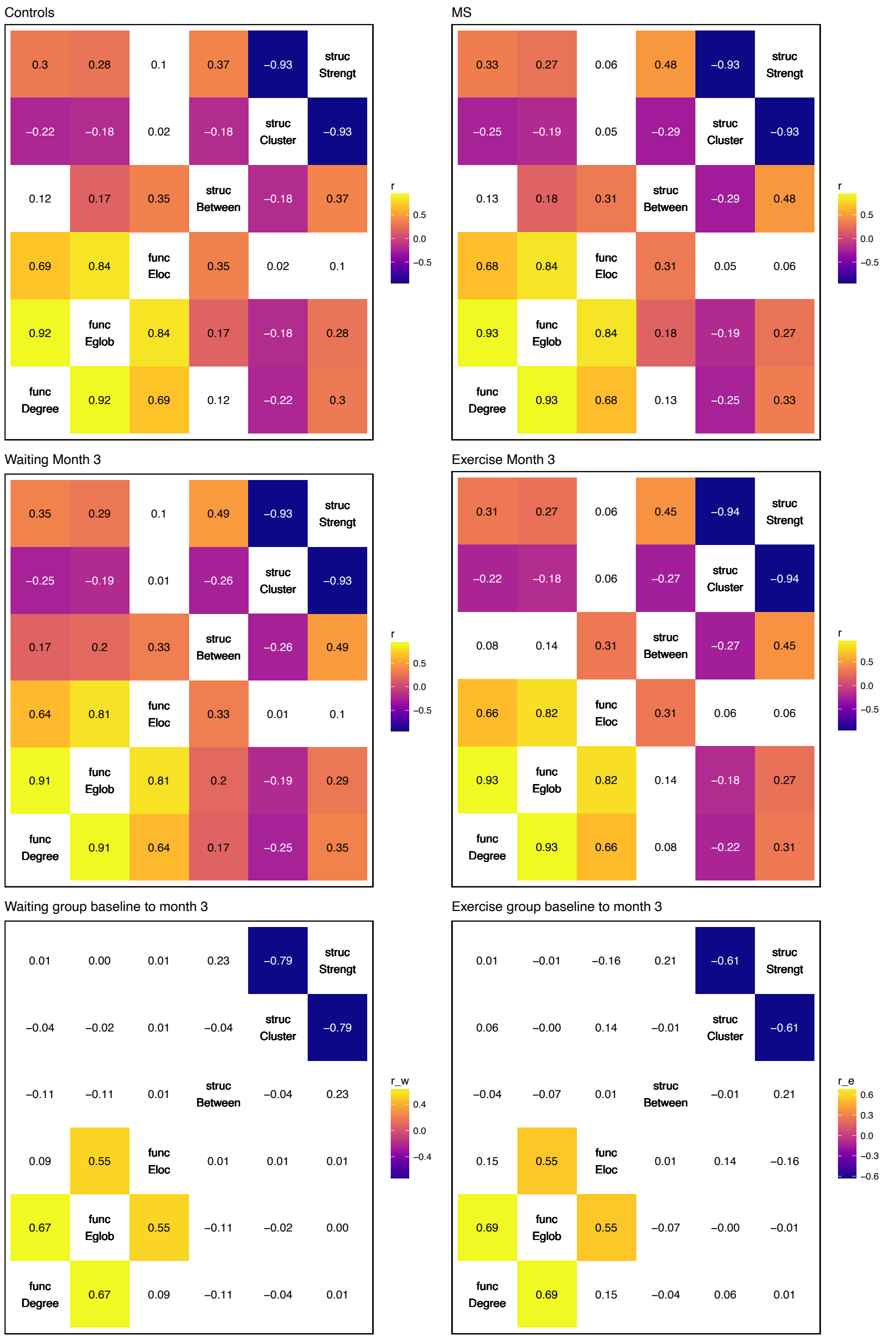

Correlations between functional and structural node metrics in healthy controls and MS patients at baseline (top) and month 3 (mid). Bottom shows the correlation between the differences from baseline to month 3 for each node metric. Colour scale indicates Pearson correlation coefficient, a white background indicates if FDR adjusted p-values were not significant.

Patterns of connectivity changes in structural connectomes

We adapted the hub disruption index further to the edge level by using mean edge values from controls as reference and plotting them against the mean values from both patient groups. For both groups we observed a significant increase in connectivity in edges with lower edge weight at baseline and a loss in connectivity for edges with high edge weights at baseline (Figure S6A) indicating a shift from hub to peripheral connectivity in MS patients. As observed for the nodal strength, we detected an increase of connectivity in the exercise group (p=0.001) compared to the waiting group during the study. The increase of local connectivity as measured by the nodal clustering coefficients corresponded with a pronounced increase in edge weight in short range connection (Figure S6B). Moreover, classifying the edges in interhemispherical, intrahemispherical and deep grey matter edges revealed a wider gap between exercise and waiting group for interhemispherical than for intrahemispherical connections, while changes for deep grey matter connections did not differ between groups (Figure S6C).

Figure S6: Structural connectivity changes on the edge level

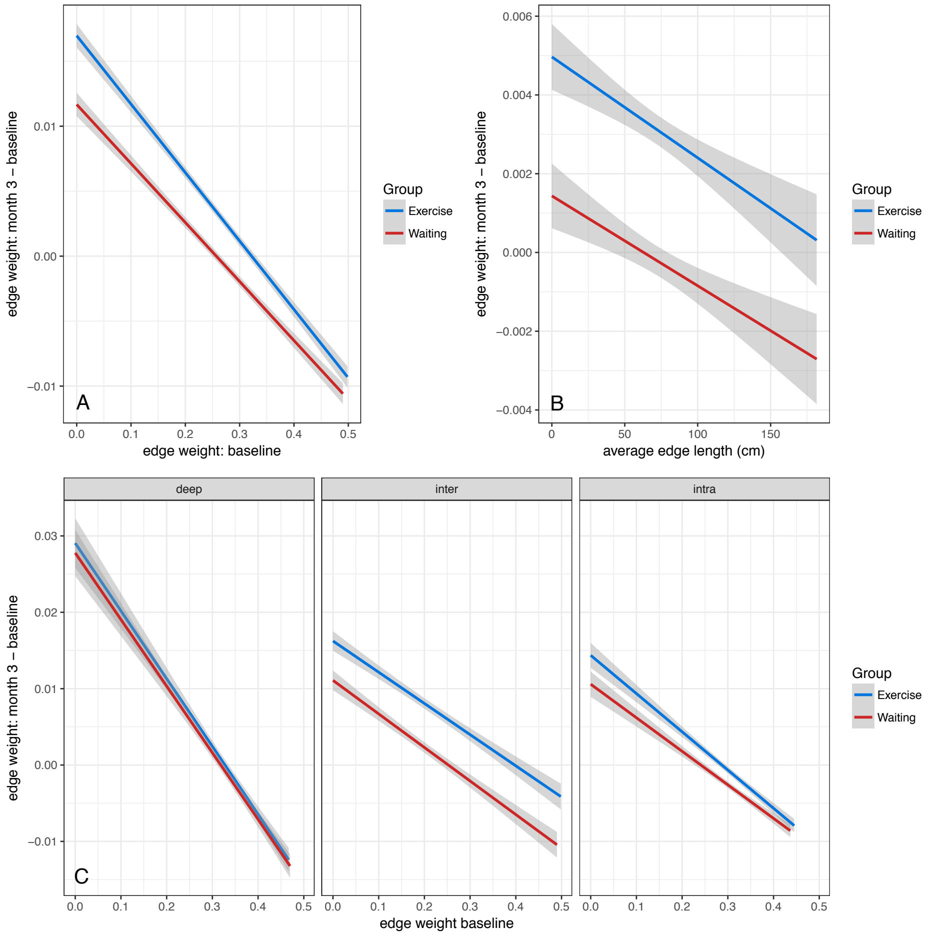

Changes in edge weights from baseline to month 3: (A) Changes plotted against the edge weight at baseline (B) Changes plotted against the edge length (Euclidian distance in cm) (C) Changes plotted against edge weight at baseline but separated in connections with deep grey matter (left), inter hemispherical connections (mid) and intrahemispherical connections (right).

Supplemental methods

Sensitivity analysis: Thresholding resting state networks

Thresholding of functional connectivity networks might affect the estimated network topology and fundamental features such as the small worldness (SR1). We followed the approach of Achard et al. and performed sensitivity analysis for threshold levels of 1% to 50% of strongest connections to proof the reliability of the selected 15% cut-off. First, we aimed to investigate if thresholding affects (I) the distribution of the degree of nodes and (II) the estimated network topology i.e. the hierarchical organization of nodes as estimated by the degree of nodes. Second, we analysed in how far different threshold might influence the main outcome of our study: the hub disruption at baseline in comparison to controls and the change in hub disruption from baseline to Month 3.

Degree distribution and network topology at different thresholds

The distribution of the degree of all nodes at all thresholds is shown in Figure S7. The median connectivity was comparable at threshold of 10% to 50% of strongest connections. Less conservative thresholds e.g. at 30 to 50% widen the spectrum only to right, which might be due to an increase in noise at less conservative thresholds. Very strict thresholds allowing only 1% or 5% of connections showed lower median connectivity and might affect fundamental network features. The findings were similar in patients and controls. Next, we investigated the correlations between the mean degree of nodes at different thresholds (Figure S8 for HC, Figure S9 for MS). The degree of a node represents the hierarchical position within a network. Thus, high correlation coefficients between the degree of nodes at different thresholds indicate a similar hierarchical organization of the networks. Even with very restrictive threshold of 1 or 5%, the correlation with other thresholds was strong ($r > 0.75$), indicating a similar topology over abroad range of thresholds. Less conservative thresholds (10 to 50%) showed very high correlations with $r > 0.99$, i.e. more or less identical topologies.

Figure S7: Distribution of the degree of nodes at different thresholds

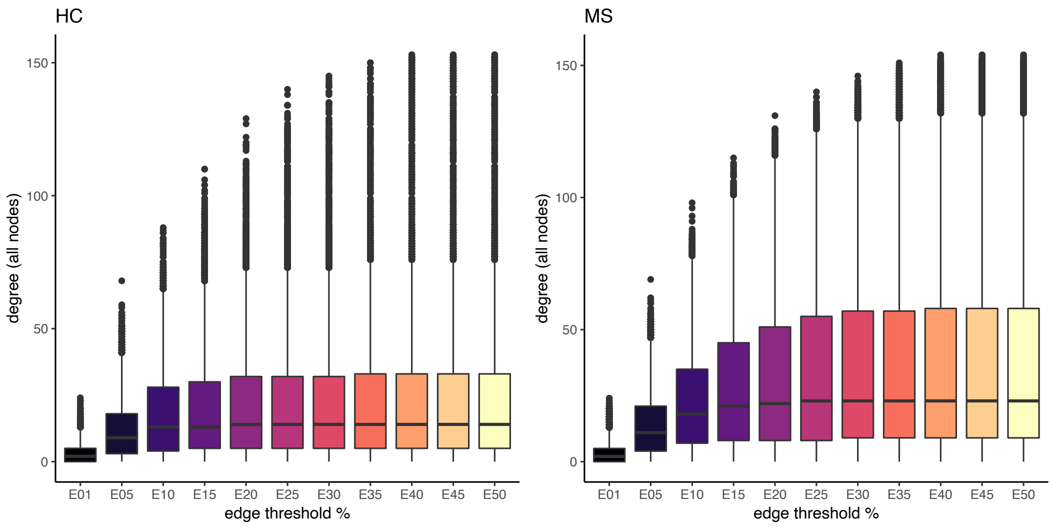

Distribution of the degree of nodes at different thresholds from 1% (E01) to 50 % of strongest connections in healthy controls (HC) and multiple sclerosis (MS)

Impact of thresholds on the hub disruption index

We used a threshold of 15% of strongest connections for our main analyses. The hub disruption index at baseline showed an increased functional connectivity in hubs of MS patients compared to controls. Applying the same analysis to thresholds from 1 to 50% (Figure S12), we observed more or less identical results in the threshold range of 10 to 50% as all regression estimates were positive and indicated increased connectivity in hubs (all $p< 0.001$). Allowing 5% of edges still showed a trend towards increased hub connectivity ($p=0.069$) and only the 1% threshold did not detect altered functional connectivity between patients and controls.

The change in hub disruption at a threshold of 15% indicated a loss of hub connectivity in the waiting group, while the exercise group showed a hub independent increase of functional connectivity. We analysed a group difference with ANOVA for all thresholds (Figure S13). Again, the 1% threshold was not able to detect a group difference. However, for all other thresholds, we observed a group difference between waiting and exercise group such as the waiting group has a hub dependent loss of connectivity (all $p < 0.001$).

Summarized, a very strict cut-off of 1 to 5% might affect the detection of fundamental features of natural networks and thus bias analyses. On the other hand, allowing more and weaker connection in the networks increases the noise in the data. Using a threshold of 15% of strongest connections seems to represent a reasonable compromise between the aim to reduce the noise in the data on one hand and to avoid the artificial affection of network topology on the other.

Figure S8: Correlation of the network topology at different thresholds in controls

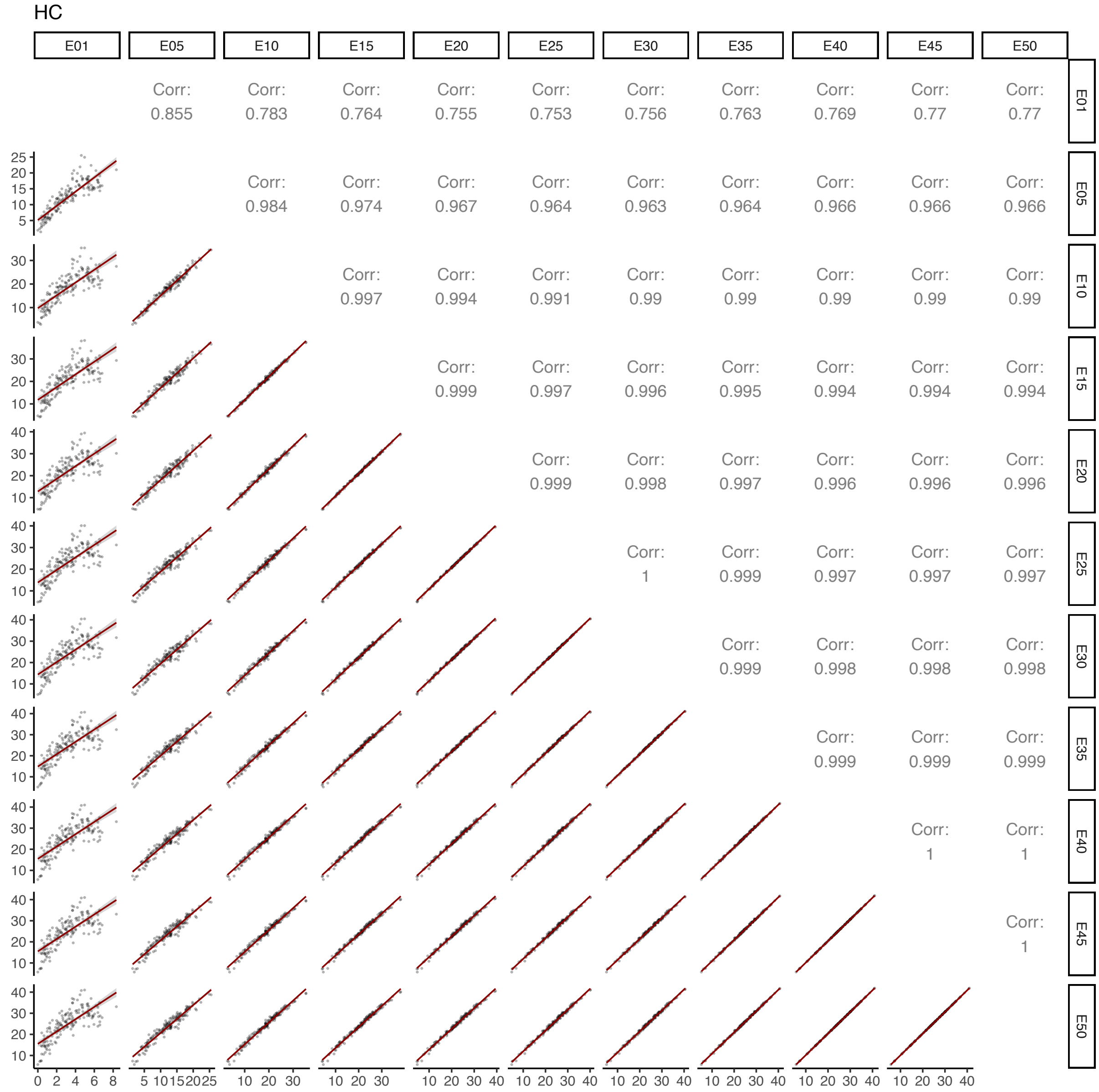

Correlation of the network topology as indicated by the degree of nodes at edge thresholds from 1% (E01) to 50% (E50) of strongest connections in healthy controls (HC): lower triangle – scatterplots with regression estimate, upper triangle – Pearson's correlation coefficient

Figure S9: Correlation of the network topology at different thresholds in MS

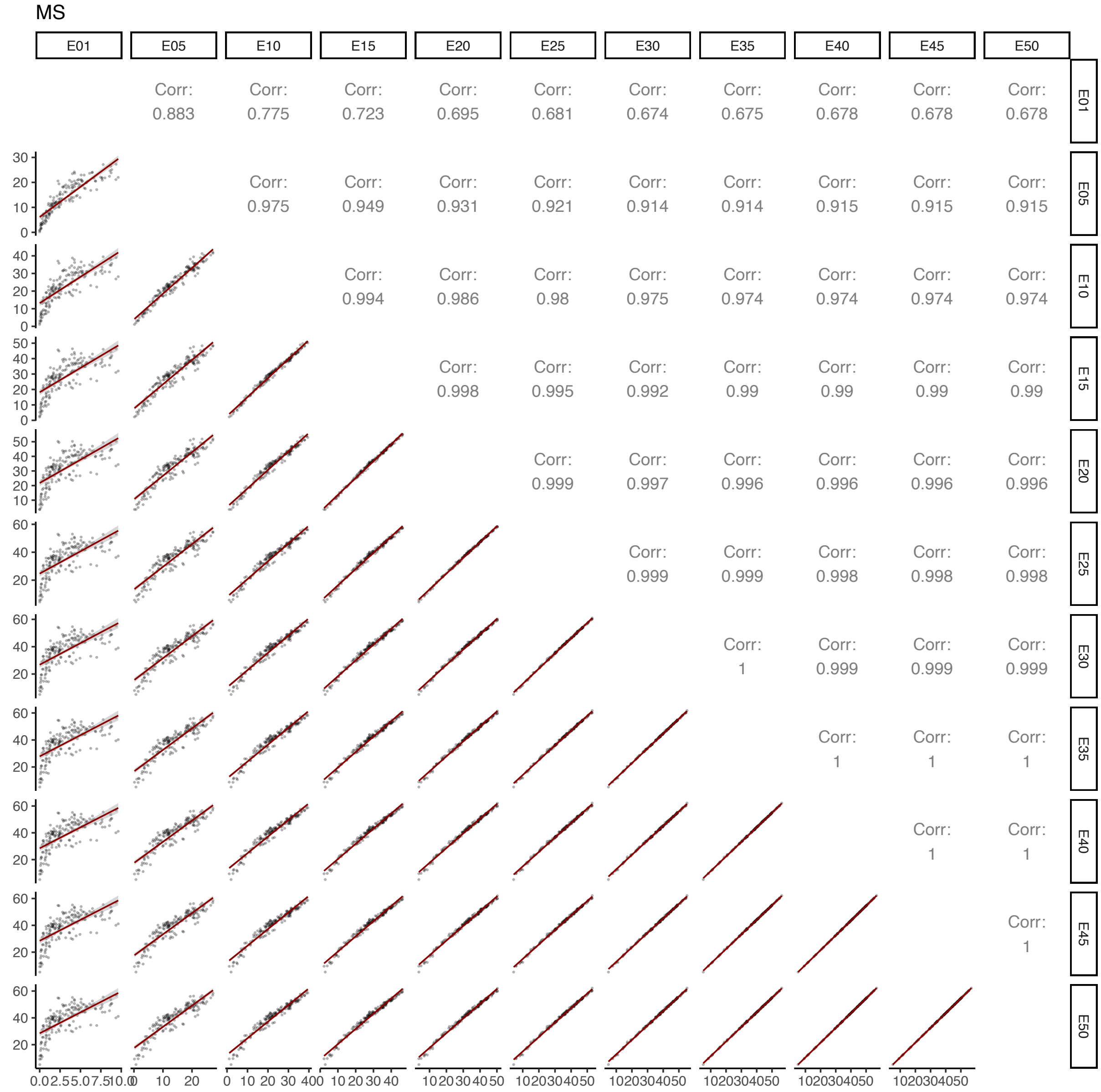

Correlation of the network topology as indicated by the degree of nodes at edge thresholds from1% (E01) to 50% (E50) of strongest connections in multiple sclerosis (MS): lower triangle – scatterplots with regression estimate, upper triangle – Pearson's correlation coefficient

Figure S10: Hub disruption index at baseline at different thresholds

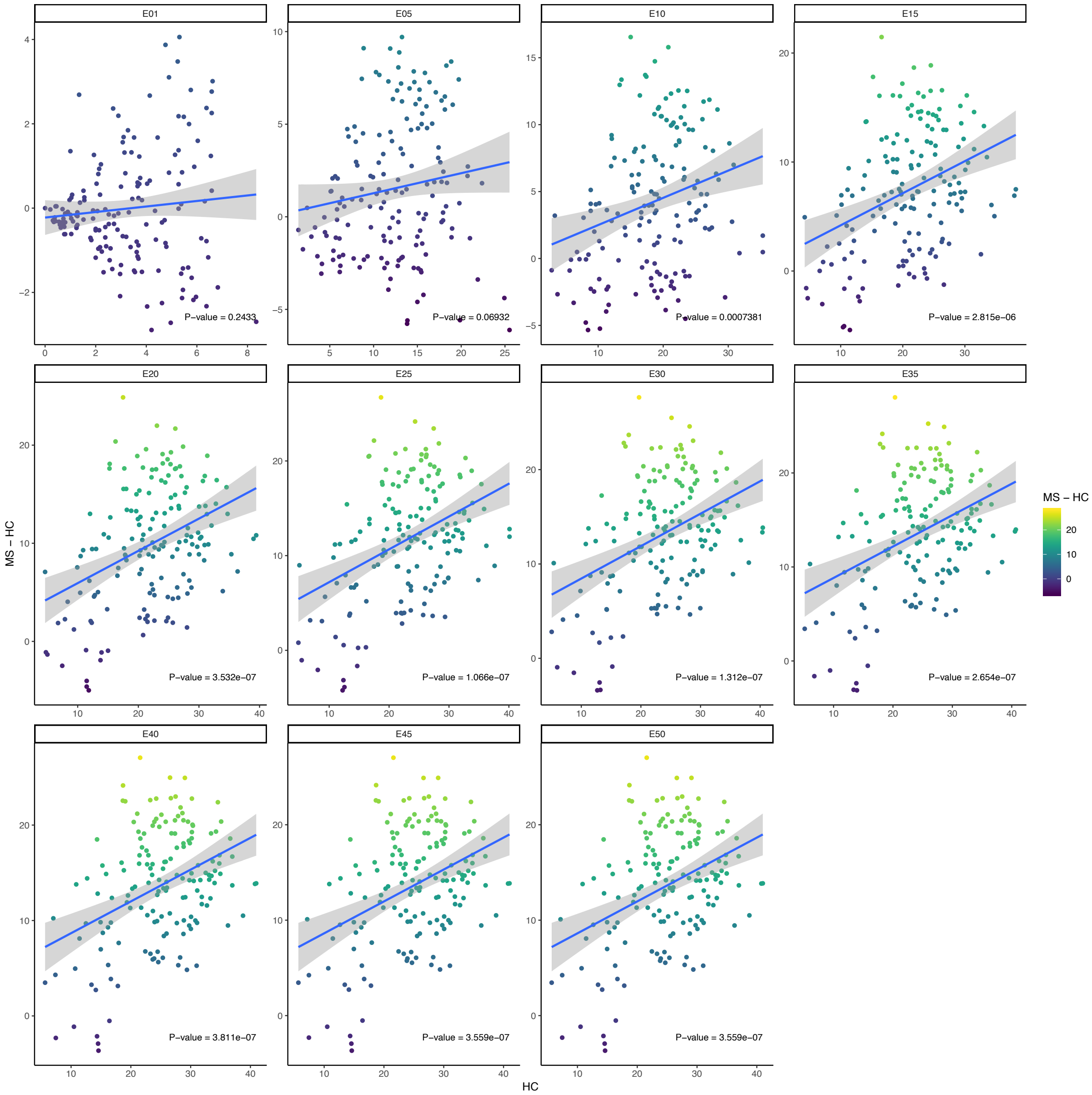

Hub disruption index at baseline for edge thresholds from 1% (E01) to 50% (E50) of strongest connections: x-axis = degree of nodes from healthy controls, y-axis = degree of nodes (multiples sclerosis, MS) minus HC

Figure S11: Hub disruption from baseline to Month 3 at different thresholds

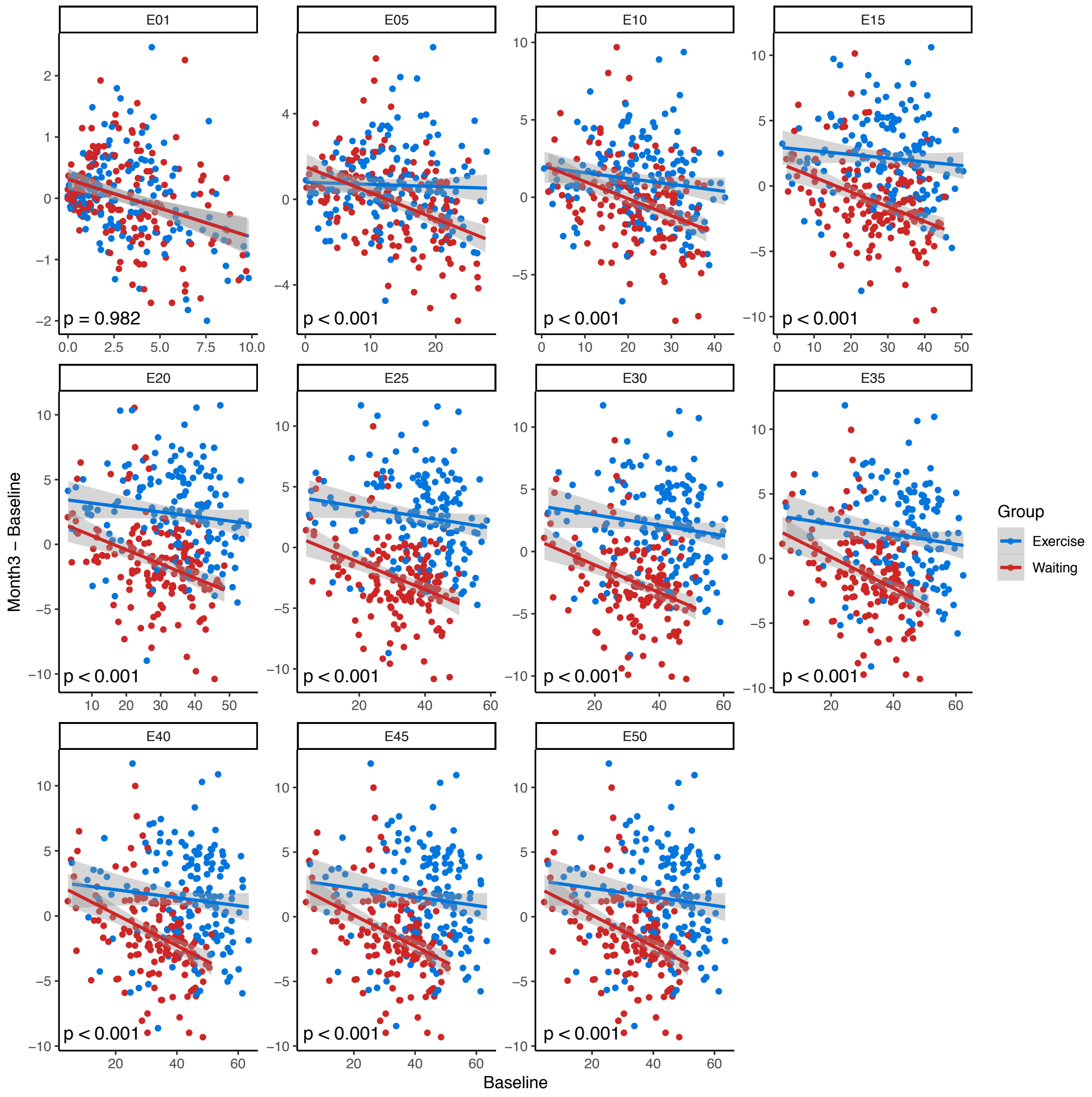

Change of hub disruption from baseline to Month 3 for edge thresholds from 1% (E01) to 50% (E50) of strongest connections: x-axis = degree of nodes from healthy controls, y-axis = degree of nodes at Month 3 minus baseline, p-values from ANOVA indicating a group difference

Supplemental references

SR1. S. Achard, R. Salvador, B. Whitcher, J. Suckling, Ed Bullmore (2006) A Resilient, Low-Frequency, Small-World Human Brain Functional Network with Highly Connected Association Cortical Hubs. *Journal of Neuroscience*, Vol. 26, N. 1, pages 63-72.
